# Dose calculation errors for volumetric modulated arc therapy plans of various complexity

**DOI:** 10.2340/1651-226X.2026.45698

**Published:** 2026-07-24

**Authors:** Emmanouil Terzidis, Fredrik Nordström, Magnus Gustafsson, Anna Karlsson, Julia Götstedt, Anna Bäck

**Affiliations:** a Department of Medical Radiation Sciences, Institute of Clinical Sciences, Sahlgrenska Academy, University of Gothenburg, Gothenburg, Sweden; b Department of Therapeutic Radiation Physics, Biomedical Engineering and Medical Physics, Sahlgrenska University Hospital, Gothenburg, Sweden

**Keywords:** Dose calculation algorithms, beam model, VMAT, dosimetric uncertainty, plan complexity

## Abstract

**Background and purpose:**

Volumetric modulated arc therapy (VMAT) plans often involve small and irregular beam apertures (i.e. high complexity), which can pose challenges for accurate dose calculations. The aim of this study was to investigate plausible dose calculation errors for VMAT plans of various complexity.

**Patient/material and methods:**

Twenty patient cases, each with three VMAT plans of different complexities (i.e. 60 plans in total), were included. All plans were calculated using six different dose calculation methods. It was assumed that greater differences between calculations reflect increased difficulty in accurately estimating dose. Basic performances of the six calculation methods were evaluated based on static fields. Three-dimensional distributions of voxel-wise two standard deviations (2SDs) in percent of local voxel dose were visually evaluated. 2SD volume histograms were analyzed as well as the mean (2SD_Mean_) and maximum 2SD within 2 cm^3^ (2SD_2cc_) for different regions of interest.

**Results:**

Higher 2SD values were generally found outside the planning target volume (PTV) compared to inside, particularly in low dose and buildup regions. Average (per treatment site) 2SD_Mean_/2SD_2cc_ values were 0.9–1.2%/1.8–5.1% in the PTV and 1.8–3.1/8.4–18.7% for the region 1 cm outside the PTV. For organs of interest, 2SD_Mean_/2SD_2cc_ values were larger than the equivalent values in the PTV, with highest values up to 5/15% observed for the prostate cases.

**Interpretation:**

Variations between dose calculation methods were larger in organs of interest than in the PTVs. Differences in 2SD distributions between the patient cases were generally larger than differences between the complexity levels.

## Introduction

Accurate and precise dose calculation is crucial for a safe and effective radiotherapy treatment. In photon radiotherapy based on a conventional linear accelerator (LINAC), dose calculation involves two key components. First is the beam model, which provides the beam fluence exiting the treatment head of the LINAC [1]. The second component is the dose calculation algorithm that uses the beam fluence to calculate the dose distribution in a given patient geometry. An accurate beam model and dose calculation algorithm are essential for predicting the dose delivered to the target volume and any nearby organs of interest (OOIs). However, clinical dose calculation methods include approximations that affect the calculated dose distribution in different ways. There are several different types of dose calculation algorithms clinically available, for example, convolution/superposition, linear Boltzmann transport equation solver, and Monte Carlo, each based on different calculation principles. Previous studies have shown that common algorithms generally agree well in homogeneous tissue regions (e.g. [2]). Discrepancies typically arise in heterogeneous regions and in regions where there is a lack of lateral charged particle equilibrium, such as in treatment plans involving small beam openings [3, 4].

Several groups have studied differences in calculated dose distributions between different clinically used dose calculation algorithms, for example, [5–10] often with particular focus on their ability to manage tissue heterogeneities. These studies demonstrate that algorithm performance can diverge considerably in regions with electron density variations. Monte Carlo methods are often regarded as the gold standard in dose calculation due to their ability to accurately simulate particle transport and radiation interactions with matter. However, even the most advanced dose calculation algorithms depend on accurate beam modeling. Treatment plans involving small beam openings or irregular beam aperture shapes (often characterized as complex plans) may require a more detailed beam model to ensure accurate and precise dose calculation [11, 12].

To our knowledge, the study from Li et al. [13] is the only one to have specifically examined how disagreement between calculation methods may relate to treatment plan complexity. Their results showed that the investigated complexity metrics had a stronger correlation with differences observed between dose distributions calculated with different calculation methods than with differences between calculated doses and diode-array detector measurements. This indicates that as plan complexity increases, variations between calculation methods become more pronounced.

The aim of this study was to evaluate 3D distributions of plausible errors in the dose calculation for volumetric modulated arc therapy (VMAT) fields of different complexities. For this purpose, the variation between different algorithms and beam models, that is, calculation methods, was studied as a measure to identify regions where accurate dose calculations are challenging.

## Patients/material and methods

### Beam models and dose calculation algorithms

To highlight regions that are more prone to calculated dose errors, different dose calculation methods with different strengths and weaknesses were used. In this study, it was assumed that larger differences between algorithms indicate greater challenges in accurately estimating the absorbed dose, which, in turn, suggests higher overall dose calculation uncertainty in those regions. Note that the aim of this work was not to determine the absolute accuracy of individual algorithms, but rather to evaluate a range of clinically realistic variations between dose calculation methods and use their in-between differences as an indicator of potential dose calculation uncertainties. This study included six dose calculation methods, implemented in two different treatment planning systems (TPSs), namely, the Eclipse (Siemens Healthineers AG, Erlangen, Germany) and RayStation (RaySearch Laboratories AB, Stockholm, Sweden). The algorithms utilized were as follows:

1. Analytical anisotropic algorithm (AAA) v16.1.0 in Eclipse
2. Analytical anisotropic algorithm (AAA) v18.1.0 in Eclipse
3. Acuros XB (AXB) v16.1.0 in Eclipse
4. Acuros XB (AXB) v18.1.0 in Eclipse
5. Monte Carlo (MC) v.3.0 in RayStation
6. Collapsed cone (CC) v.5.8 in RayStation

All beam model configurations used in this work were created for a 6 MV photon beam from a Varian TrueBeam linac with a Millenium MLC (Siemens Healthineers AG, Erlangen, Germany) and are intended for clinical application. Although not all of them are currently in clinical use. Besides MLC-specific measurements, the beam configurations in Eclipse (i.e. both versions of AAA and AXB algorithms) share the same general commissioning data (e.g. depth doses, profiles, and output factors). AAA version 16.1.0 is the algorithm currently in clinical use. The main difference between version 18.1.0 of AAA and AXB and the earlier 16.1.0 version is an enhanced modeling of the MLC [14] in version 18.1.0. Values of MLC-specific parameter values as well as effective target spot sizes used for the different beam models and algorithms are presented in Table S1a in the supplementary material.

The beam models for the MC and CC algorithms in RayStation were commissioned using a different set of in-house measurement data (measured later in time, but evaluated to be in good agreement, compared to that used for Eclipse configurations) in combination with output factors from Varian reference beam data. Comparisons between Varian reference data and measured data were in good agreement; for example, all depth doses and 96% of lateral profiles were within 95% gamma pass rate using 2%/2 mm criteria. The MLC parameters were acquired in accordance with Saez et al. [15], and primary source width was optimized using profile measurements for a 0.5 × 0.5 cm^2^ beam. Information regarding MLC-specific parameters and primary source widths for the beam models in RayStation can be found in Table S1b in the supplementary material.

For all dose calculation methods, a 1 mm dose grid resolution was utilized. The spacing between CT slices was 2–3 mm. In this study, the focus was placed on calculated dose discrepancies associated with complex treatment plans rather than those arising from heterogeneities within the patient volume. For this reason, the electron and mass density for all voxels in the patient’s CT data were set to 1, and the material was set to water. To be able to do a voxel-wise comparison between all dose distributions, some of the dose distributions had to be resampled using trilinear interpolation, to spatially match the AAA v16.1.0 dose distributions. Monte Carlo calculations in RayStation were performed with a statistical uncertainty of 0.1%.

### Dose calculations for static fields

Before investigating the variation of different dose calculation methods for VMAT treatment plans, some general aspects of those differences were studied. For this purpose, a number of static fields were created and calculated for 6 MV photons from the Varian TrueBeam linac. The first consisted of a 10 **×** 10 cm jaw-defined field with the MLC fully retracted and using a source to surface distance (SSD) of 90 cm. This was used to verify that all methods gave the same results in a reference situation. The second field included multi-leaf collimator (MLC) with leaf protrusions as illustrated in Figure 2, using the same jaw positions as the first field. These MLC protrusions were meant to test the performance of the different dose calculation methods specifically when it comes to the MLC field edges and the modeling of the MLC. Differences between dose calculation methods for the two static fields were visually evaluated by extracting lateral dose profiles in the isocenter plane at 10 cm depth. The open field was further evaluated for the different calculation methods by calculating the standard deviation using a coverage factor with *k* = 2 (2SD) of the absorbed dose in the isocenter point. A higher value of 2SD means a larger difference between different algorithms, which, in this work, is assumed to indicate a higher risk for dose calculation errors. The deviation of the physical field sizes (i.e. both X and Y directions) from the nominal value (i.e. 10 **×** 10 cm) was also assessed for the different calculation methods. For the static field with MLC protrusions, the 2SD of dose was calculated on a voxel-by-voxel basis across the dose distributions calculated by the six dose calculation methods, resulting in a three-dimensional (3D) distribution of standard deviations. This 3D 2SD map quantified the variance in calculated dose arising from differences between the six dose calculation methods. From this point onward, reference dose refers to the dose calculated by AAA v16.1.0 for each plan since this was the calculation method used in clinical routine. Voxels receiving less than 5% of the reference dose were excluded from this analysis.

The variation between the different dose calculation methods in field size dependence was evaluated by the 2SD of the dose in the isocenter point for MLC-defined fields of decreasing size ranging from 12 **×** 12 cm down to 1 **×** 1 cm, with the jaws positioned 2 cm behind the MLC leaf-tips. Additionally, off-axis effects were investigated by positioning the center of a MLC-defined 3 **×** 3 cm field at lateral offsets of 2, 4, 6, 8, 10, and 12 cm from the isocenter toward the X2 jaw and by evaluating the 2SD of the dose at 10 cm depth in the center of the fields. The jaws were again positioned 2 cm behind the MLC leaf-tips. In all cases, the evaluations were done at 10 cm depth with the SSD set to 90 cm at the central axis. Each field was assigned 240 monitor units (MUs) for the dose calculations, corresponding to 2 Gy at isocenter in the calibration geometry (i.e. 10 **×** 10 cm field, SSD = 90 cm at 10 cm depth in water).

### Dose calculations for treatment plans

In total, 60 VMAT plans were included. The plans were based on CT data and clinical segmentations from 20 cancer patients: five prostate, five head and neck, five lung, and five gynecological cases. The plans were initially optimized and calculated using 6 MV photons with the AAA v16.1.0 dose calculation algorithm (1 mm dose grid). All treatment plans were planned on a Varian TrueBeam linac with the Millenium MLC. Jaw tracking was used for all plans with the exception of prostate cases #1 and #5. Additional information regarding treatment planning parameters for the included VMAT plans can be found in the Supplementary Tables S2–S5.

For each case, a simple and a complex plan were created in addition to the clinical plan, following the methodology described in our previous work [16]. For the simple plans, there was a reduction in MU ranging from 1.3 to 42.6% compared to the respective clinical plan. For the complex plans, there was an increase in MU ranging from 7.4 to 167.4%. Each of the 60 VMAT plans (20 clinical, 20 simple, and 20 complex) was calculated with the six dose calculation methods.

3D 2SD maps were generated for the 60 VMAT plans. Differential 2SD volume histograms were generated from the 3D 2SD distributions for all 60 VMAT plans. These were calculated for the planning target volume (PTV) and a single OOI per treatment site, that is, the rectum for prostate and gynecological cases, the contralateral parotid gland for head and neck cases, and the spinal cord for lung cases. These OOIs were selected as they are considered of high priority for the specific cases that were investigated. Wilcoxon signed-rank tests were performed on pairs of 2SD histogram distributions to determine whether there were significant differences (i.e. *p* < 0.05) in calculated dose variation between plans of different complexity (i.e. clinical vs. simple plans and clinical vs. complex plans). Finally, the maximum 2SD for a volume of 2 cm^3^ (2SD_2cc_) and the mean value of 2SD (2SD_Mean_) were calculated inside the PTV, in the region 1 cm outside the PTV and in one OOI per treatment site as described earlier. The region 1 cm outside the PTV was chosen, since it was found to be a region of interest in a previous study, where delivery variations were investigated [16].

## Results

**Figure 1:**
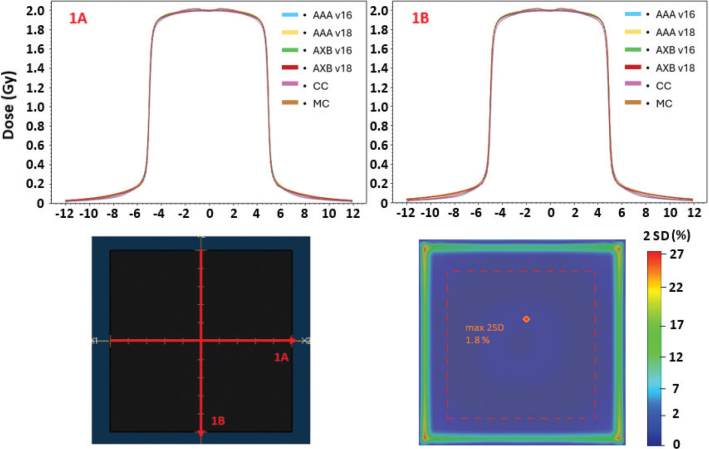
First row: dose profiles (1A, 1B) from a static 10 × 10 cm jaw-only field calculated using six methods. Field properties: 240 monitor units, 10 cm depth, source-to-surface distance 90 cm. Profiles 1A and 1B correspond to the x- and y-axes, respectively, according to the IEC 61217 coordinate system. Bottom row: (1) profile positions in jaw-only field (left), and (2) 2D map at 10 cm depth of the 2‑standard‑deviation in % of the local dose between the different dose calculation methods for the open field (right). The red point illustrates the point with the highest 2SD value within the 8 × 8 cm^2^ central part of the beam, which was 1.8% and located at 0.6 and 1.7 cm off-axis in the x and y directions, respectively.

**Figure 2:**
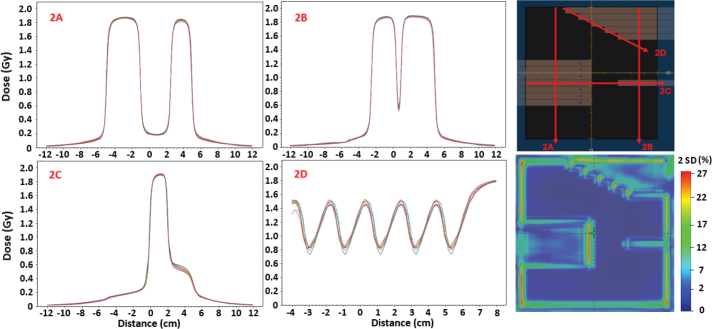
Dose profiles (2A-2D) from a static 10 × 10 cm jaw field with multileaf collimator (MLC), calculated using six methods. Field properties: 240 monitor units, 10 cm depth, source-to-surface distance 90 cm. Profiles 2A and 2B in the y-direction and 2C in the x-direction, according to the IEC 61217 coordinate system. Rightmost column: (1) illustration of field geometry and profile positions (top) and (2) 2D map at 10 cm depth of the 2‑standard‑deviation in % of the local dose between the different dose calculation methods (bottom).

**Figure 3:**
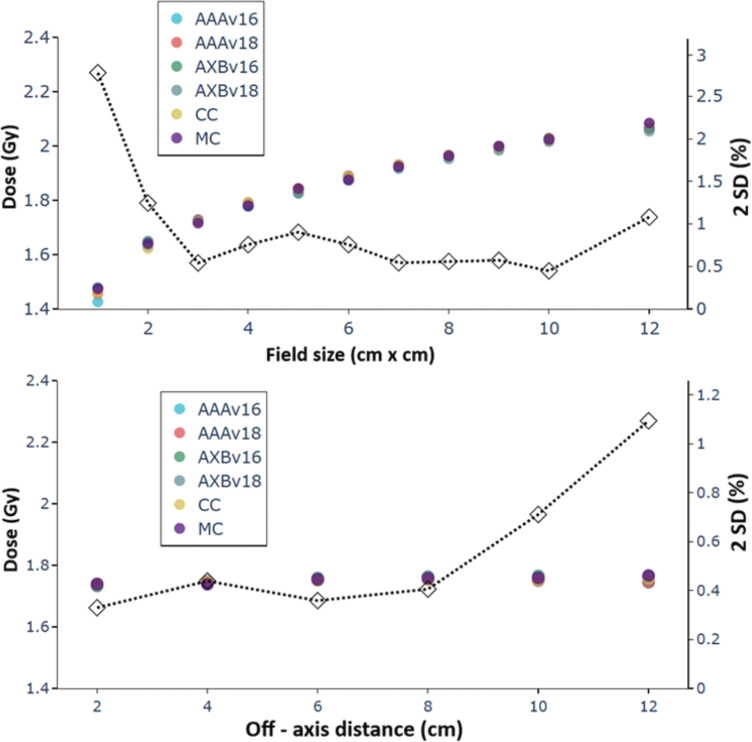
(Upper) Isocenter doses for static square fields of various sizes using different dose calculation methods. (Lower) Dose at 10 cm depth in the center of a 3 × 3 cm field for varying lateral shifts from isocenter toward the X2 jaw. All fields were calculated with 240 MU and 90 cm SSD. 2 standard deviations (2SD) in % of the local dose between calculation methods at each point is indicated by the diamond markers connected with a dotted line.

Visual evaluation of lateral profiles for a reference situation of a 10 × 10 cm^2^ jaw-defined open square field demonstrated consistency across the different calculation methods (Figure 1). The 2SD of the calculated doses in the isocenter was less than 0.5% of the local reference dose, and the field sizes agreed within 0.09 cm of the nominal 10 cm. The highest 2SD for the open part of the field (i.e. within the 8 × 8 cm^2^ central part of the field) was 1.8%. This was found in the position at 0.6 and 1.7 cm off axis in the x and y directions, respectively (Figure 1). For the field including MLC protrusions, differences between calculation methods were most pronounced in the MLC-blocked part of the beam near the MLC leaf-tip and in regions of high-dose gradients (Figure 2). For field sizes above 1 × 1 cm^2^, the 2SD values for the isocenter dose were below 1.3% of the local reference dose, and it was 2.8% for the 1 × 1 cm^2^ field (Figure 3). For off‑axis fields, the 2SD values in the central points were below 0.5% at off‑axis distances less than 10 cm, reaching up to 1.1% at 12 cm off-axis.

**Figure 4:**
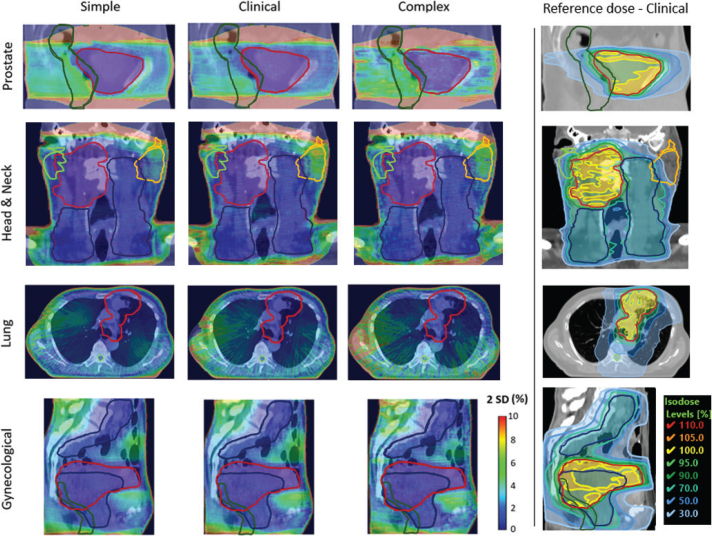
Representation of voxel-wise 2 standard deviations (2SD, SD with *k* = 2) distributions for one case each, prostate (plan #1), lung (plan #2), head & neck (plan #2), and gynecological (plan #3) divided between simple, clinical, and complex plans. For each voxel, the 2SD of dose for six different dose calculation algorithms normalized to the local dose is shown. Voxels with reference dose less than 5% were excluded. For each treatment site, a central slice through the planning target volume (PTV) is depicted. Coronal views are shown for head & neck cases, transversal for the lung cases, while sagittal views are presented for prostate and gynecological cases. The red contours refer to the PTVs of the primary tumors, and the blue contours indicate the PTVs of the lymph nodes. The dark green contours refer to rectum for the prostate and gynecological cases. For the lung case, the spinal cord is depicted with a light green contour. For the head & neck case, the light green and orange contours refer to the ipsilateral and contralateral parotids, respectively. The reference dose distribution for each clinical case is shown in the rightmost column.

**Figure 5:**
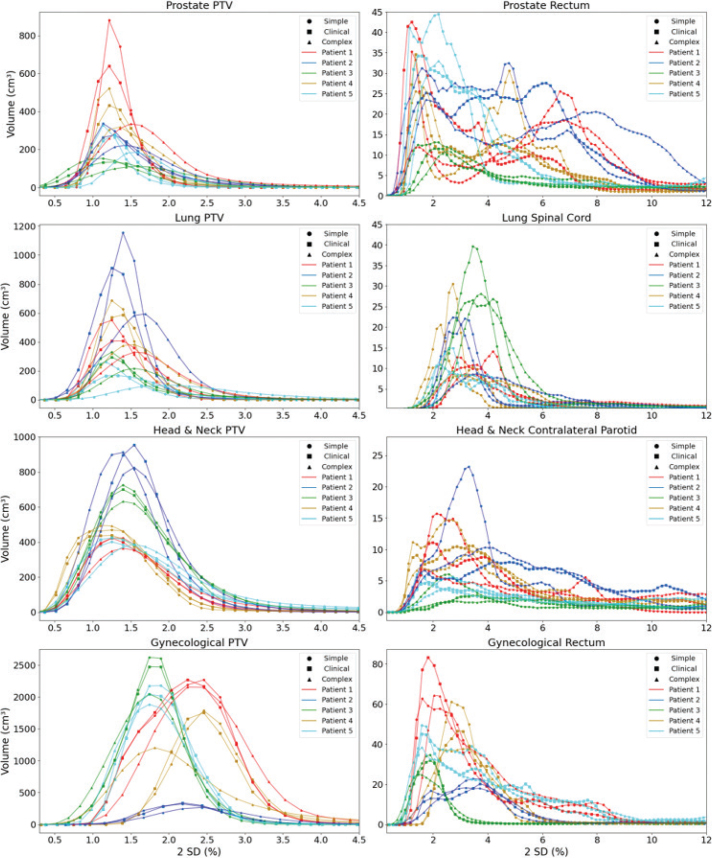
Two standard deviation (2SD, SD with *k* = 2) differential histograms for all cases and plans grouped by four different treatment sites. The bin widths for x-axis were 0.1% 2SD. For prostate and lung cases, the PTV encompasses the primary tumor only, whereas for head & neck and gynecological cases, lymph node PTVs are included when applicable.

The higher 2SDs were generally observed outside the PTV volume and the highest in the low dose and build up regions (Figure 4). Figure 5 presents the differential 2SD volume histogram curves for PTVs and selected OOIs across the various plan complexities for all cases studied. The histograms demonstrated that the differences between patient cases were generally larger than differences between complexity levels. Statistically significant differences in 2SD histograms between clinical and simple plans were observed in 11 out of 20 cases for PTV and in 7 out of 20 cases for the selected OOIs (Table 1). For comparisons between clinical and complex plans, significant differences were found in 15 out of 20 cases for PTV and 8 out of 20 for OOIs. As shown in Table 2, both 2SD_Mean_ and 2SD_2cc_ were generally higher for the OOIs compared to the inside of the PTV. For the OOIs, the highest 2SD_Mean_ values were observed for the prostate and head & neck cases, while the 2SD_2cc_ values for the OOIs were highest for the rectums in the prostate and gynecological cases. The 2SD_Mean_ and 2SD_2cc_ values inside the PTV were similar across all treatment sites, except head & neck, with ranges of 0.9–1.2% and 1.8–2.9% respectively. For the head & neck 2SD_2cc_ values, it was up to 5.1%. The 2SD_Mean_ and 2SD_2cc_ values for the region 1 cm outside the PTV were generally larger (highest values 3.1 and 18.7%, respectively) than the corresponding values for the PTVs. Overall, the results showed that the influence of plan complexity on dose calculation variations was case‑dependent.

**Table 1:** *P*-values from Wilcoxon signed-rank tests comparing the standard deviation with *k* = 2 (2SD) distribution between the clinical and simple version and between the clinical and complex version for each case. PTV and one organ of interest (OOI) were analyzed: rectum (prostate, gynecological), spinal cord (lung), contralateral parotid (head & neck).

Case	PTV: Clinical vs. simple	PTV: Clinical vs. complex	OOI: Clinical vs. simple	OOI: Clinical vs. complex
Prostate #1	< 0.05	< 0.05	0.77	0.49
Prostate #2	< 0.05	< 0.05	0.05	0.11
Prostate #3	0.14	< 0.05	0.31	0.49
Prostate #4	0.10	< 0.05	0.69	0.31
Prostate #5	0.13	< 0.05	< 0.05	0.52
Head & Neck #1	< 0.05	< 0.005	0.06	< 0.05
Head & Neck #2	< 0.05	0.92	< 0.05	0.53
Head & Neck #3	< 0.05	< 0.05	0.40	< 0.05
Head & Neck #4	0.17	< 0.05	< 0.05	< 0.05
Head & Neck #5	< 0.05	< 0.05	< 0.05	0.20
Lung #1	< 0.05	< 0.05	< 0.05	< 0.05
Lung #2	< 0.05	< 0.05	0.59	< 0.05
Lung #3	0.24	0.29	0.42	< 0.05
Lung #4	0.35	< 0.05	< 0.05	< 0.05
Lung #5	< 0.05	< 0.05	0.35	< 0.05
Gynecological #1	< 0.05	< 0.05	0.85	0.21
Gynecological #2	0.19	0.06	0.19	0.10
Gynecological #3	0.48	0.18	0.08	0.06
Gynecological #3	< 0.05	0.85	0.92	0.95
Gynecological #3	0.31	< 0.05	< 0.05	0.16

PTV: planning target volume; OOI: one organ of interest.

PTV and OOI were analyzed: rectum (prostate, gynecological), spinal cord (lung), and contralateral parotid (head & neck).

**Table 2:** Average values (three cases per treatment site and complexity level) for the maximum standard deviation with *k* = 2 (2SD) to a volume of 2 cm^3^ (2SD_2cc_) and the mean value of 2SD (2SD_Mean_) inside the PTV, in the region 1 cm outside the PTV as well as for one organ (OOI) at risk per treatment site: rectum (prostate, gynecological), spinal cord (lung), and contralateral parotid (head & neck).

Prostate		Inside PTV	1 cm outside PTV	OOI
Average 2SD_2cc_ (Local %)	Simple	1.9	9.7	14.6
	Clinical	1.9	11.2	14.8
	Complex	2.8	13.1	15.0
Average 2SD_Mean_ (Local %)	Simple	0.9	2.4	4.2
	Clinical	1.0	2.3	4.4
	Complex	1.2	3.1	5.0

Head & Neck		Inside PTV	1 cm outside PTV	OOI

Average 2SD_2cc_ (Local %)	Simple	4.5	18.0	7.8
	Clinical	4.8	18.3	9.1
	Complex	5.1	18.7	9.3
Average 2SD_Mean_ (Local %)	Simple	1.1	2.0	3.3
	Clinical	1.2	2.2	4.3
	Complex	1.2	2.4	4.7

Lung		Inside PTV	1 cm outside PTV	OOI

Average 2SD_2cc_ (Local %)	Simple	1.8	10.2	7.2
	Clinical	2.1	13.3	7.2
	Complex	2.9	12.6	8.9
Average 2SD_Mean_ (Local %)	Simple	0.9	1.9	2.8
	Clinical	1.0	2.2	2.8
	Complex	1.2	2.6	3.6

Gynecological		Inside PTV	1 cm outside PTV	OOI

Average 2SD_2cc_ (Local %)	Simple	2.1	8.4	13.3
	Clinical	2.3	9.4	14.8
	Complex	2.5	10.5	14.8
Average 2SD_Mean_ (Local %)	Simple	1.1	1.8	2.9
	Clinical	1.1	1.9	3.2
	Complex	1.1	2.0	3.3

PTV: planning target volume; OOI: one organ of interest.

## Discussion and conclusion

Variations in dose distributions calculated using six different methods were investigated for static fields and VMAT plans of various complexity and treatment sites. In the static fields, higher 2SDs were observed for regions close to the MLC leaf-tips, in smaller fields and at larger off axis positions. For VMAT plans, dose variations were generally higher in regions outside the PTV compared to inside the PTV. Dose variations in the OOIs were larger than within the PTV, although the magnitude varied between patient cases and treatment sites. Variations between patient cases were generally larger than variations between complexity levels. Identifying regions where beam models and calculation algorithms diverge provides valuable insight into which anatomical regions are more prone to dose calculation uncertainties and where clinical dose estimates may be less reliable.

The results for the 10 × 10 cm^2^ jaw-defined static open field showed that the 2SDs of the calculated doses were larger off axis compared the center, that is, within 0.5% in the center and 1.8% within the 8 × 8 cm^2^ central part of the beam. However, the 2SDs in the static field with MLC protrusions were much larger, especially close to the MLC leaf-tip, which indicates that the off-axis variations were less dominant in the MLC field. Based on the results obtained for the static fields, it was anticipated that VMAT treatment plans with complex aperture shapes and high beam modulation would exhibit larger dose calculation variations. Hence, higher 2SD_Mean_ and 2SD_2cc_ values were expected for complex plans compared to clinical and simple plans. However, the difference in average values per treatment site was within 0.9 and 1.7% for the PTV and OOIs, respectively (Table 2). Statistically significant differences in 2SD histograms between treatment plans of different complexity levels were observed in several cases (Table 1). Differences between complexity levels were generally smaller than those between individual patient cases.

The evaluations for the open static field were consistent with the findings of Rostami et al. [7], who performed a comparative evaluation of dose calculation methods (i.e. AAA and AXB (v.16.1) in Eclipse and CC and MC in RayStation) similar to those used in the present study. Similar to their findings, for an open 10×10 cm² field, the dose difference between different algorithms in the isocenter was less than 1%. In addition, our study extended the evaluation to include static fields with MLC protrusions as well as VMAT treatment plans. It is worth noticing that Rostami et al. also investigated different beam energies, flattening-filter-free beams, and varying SSDs. These factors are not included in this study but are potentially relevant, given their dependence on the beam model.

Li et al. [13] investigated how discrepancies between different dose calculation algorithms relate to treatment plan complexity for Intensity Modulated Radiotherapy (IMRT). They evaluated 53 complexity metrics and found that discrepancies among CC, MC, and AAA algorithms correlated with some of these metrics. In their analysis, metrics related to plan modulation (e.g. variation in MLC speed and acceleration) were found to have stronger correlations with differences between dose calculation algorithms than metrics related to the aperture shape, for example, the edge area metric (EAM) [17, 18]. In the present study, VMAT plans were divided into different complexity levels. It has been shown in a previous study that significant differences in EAM values exist between these complexity levels [16]. In this study, several statistically significant differences were found in the SD histograms between plans of different complexity levels.

The number of dose calculation algorithms included in this study was relatively small, which limits the strength of the standard deviation estimated in each voxel. This limitation could be addressed by effectively increasing the sample size through systematic perturbations of the existing beam configurations, for example, by introducing small variations to parameters such as the dosimetric leaf gap (DLG) or leaf transmission. As demonstrated by former studies, for example [19, 20], even an offset in the DLG as small as 1 mm can result in notable dose variations in VMAT plans.

In this study, only dose variations related to the calculation method were considered. Incorporating additional sources of uncertainty, such as those related to treatment delivery, would allow for a more complete understanding of how different factors contribute to the overall accuracy of radiotherapy dose estimation. In this context, our group has previously investigated delivery-related variations by introducing systematic and random offsets of treatment machine parameters, to simulate potential deviations during treatment delivery [16]. Results from that study showed that 2SDs within the CTV were larger for plans of higher complexity, highlighting that they are more sensitive to delivery variations. This was also the case in the current study where differences between dose calculation methods were observed between plans of different complexity for the PTV. Both studies, however, suggest that increased variations can mainly be observed in regions outside the border of the PTV, highlighting these regions as more sensitive to both calculation and delivery variations. Future analyses combining both calculation and delivery uncertainties could offer valuable insights into the cumulative effects that influence treatment quality in advanced radiotherapy.

The different calculation methods agreed well for open static fields, but larger variations were found in regions near the MLC leaf-tips, in small fields (< 2 cm) and at large off axis positions (≥ 10 cm). For VMAT plans, variations between different dose calculation methods were larger in OOIs, where 2SDs larger than 10% (normalized to local dose) were found in some cases compared the PTVs, where the 2SDs were most often within 4%. Differences between patient cases were generally larger than differences between complexity levels. These findings suggest that inter algorithm differences, thus the estimated dose calculation uncertainty, are more strongly influenced by patient-specific factors than by plan complexity.

## Supplementary Material



## Data Availability

Due to patient privacy restrictions, the datasets generated and/or analyzed in this study are not publicly available. However, parts of the data could be provided by the corresponding author upon reasonable request.
