# Supplementary material for: Dose calculation errors for volumetric modulated arc therapy plans of various complexity
Source: Acta Oncol. 2026 Jul 24;65:45698. doi: 10.2340/1651-226X.2026.45698 (PMC13410304; doi:10.2340/1651-226X.2026.45698)
Supplement: Supplementary file 1 [file AO-65-45698-s1.pdf]

Supplementary material has been published as submitted. It has not been copyedited, or typeset by Acta Oncologica

Dose calculation errors for volumetric modulated arc therapy plans of various complexity

Emmanouil Terzidis[1,2], Fredrik Nordström[1,2], Magnus Gustafsson[2], Anna Karlsson[1,2], Julia Götstedt[1,2], Anna Bäck[1,2]

[1]Department of Medical Radiation Sciences, Institute of Clinical Sciences, Sahlgrenska Academy, University of Gothenburg, Gothenburg, Sweden

[2]Department of Therapeutic Radiation Physics, Medical Physics and Biomedical Engineering, Sahlgrenska University Hospital, Gothenburg, Sweden

Correspondence: Emmanouil Terzidis, Department of Medical Radiation Sciences, Institute of Clinical Sciences, Sahlgrenska Academy, University of Gothenburg, Gothenburg, Sweden, Gula Stråket 2B, 413 46, Gothenburg, Sweden. Email: emmanouil.terzidis@gu.se

Supplementary material

Supplementary Table S1a: Model configuration parameters for 6 MV in Eclipse

**Algorithm**	**DLG [cm]**	**Transmission**	**Effective spot size in X [mm]**	**Effective spot size in Y [mm]**
**AAA v16.1.0**	**0.1550**	**0.0152**	**0**	**0**
**AAA v18.1.0**	**-0.043945**	**0.014589**	**0**	**0**
**AXB v16.1.0**	**0.1550**	**0.0152**	**1**	**1**
**AXB v18.1.0**	**-0.043937**	**0.014589**	**0.5**	**0.7**

Supplementary Table S1b: Model configuration parameters for 6 MV in RayStation

**Algorithm**	**Tongue and groove [cm]**	**Leaf tip width [cm]**	**Leaf tip transmission**	**Corner transmission**	**Primary source X-width [cm]**	**Primary source Y-width [cm]**
**Collapsed Cone**	**0.044**	**0.444**	**0.195**	**0.56155**	**0.07**	**0.07**
**Monte Carlo**	**0.044**	**0.444**	**0.195**	**0.56155**	**0.035**	**0.035**

Supplementary Table S2: Treatment plan information for all prostate cases. Treatment plans characterized as simple, and complex have been derived from the clinical plan for each case by altering the Monitor Unit (MU) optimization options and the Aperture Shape Controller (ASC) settings.

	Prescribed dose (Gy)	Number of fractions	Arc geometry	Total MU	MU Optimization Min/Max/Strength	ASC
Prostate 1 simple			2 full*	351.5	50/400/100	Very High
Prostate 1 clinical	70	35		434.1	50/550/75	Very Low
Prostate 1 complex				930.2	900/1300/100	Off
Prostate 2 simple			2 full*	700.3	50/700/100	Very High
Prostate 2 clinical	66	22		1003.3	70/1000/75	Very Low
Prostate 2 complex				1527.6	1400/1800/100	Off
Prostate 3 simple			2 full*	800.1	70/800/100	Very High
Prostate 3 clinical	66	22		1108.7	70/1000/75	Very Low
Prostate 3 complex				1882.2	1400/2000/100	Off
Prostate 4 simple			2 full*	426.2	50/450/100	Very High
Prostate 4 clinical	70	35		553.3	50/550/75	Very Low
Prostate 4 complex				962.3	1000/1400/100	Off
Prostate 5 simple			2 full*	554.1	50/500/100	Very High
Prostate 5 clinical	66	22		665.2	50/600/75	Very Low
Prostate 5 complex				1198.3	1500/1900	Off

* one counter clockwise arc (179°-181°) and one clockwise (181°-179°)

Supplementary Table S3: Treatment plan information for all head & neck cases. Treatment plans characterized as simple, and complex have been derived from the clinical plan for each case by altering the Monitor Unit (MU) optimization options and the Aperture Shape Controller (ASC) settings.

	Prescribed dose (Gy)	Number of fractions	Arc geometry	Total MU	MU Optimization Min/Max/Strength	ASC
Head & neck 1 simple			2 full	352.3	50/35/100	Very High
Head & neck 1 clinical	68/52.1	34		495	0/600/50	Very Low
Head & neck 1 complex				1058.8	1000/2000/100	Off
Head & neck 2 simple				351.1	10/350/100	Very High
Head & neck 2 clinical	68/52.1	34	2 full	611.2	70/700/50	Off
Head & neck 2 complex				1171.1	1000/2000/100	Off
Head & neck 3 simple				421.3	10/420/100	Very High
Head & neck 3 clinical	68/52.1	34	2 full	601.2	0/600/50	Very Low
Head & neck 3 complex				1111.4	1000/2000/100	Off
Head & neck 4 simple				451	50/450/100	Very High
Head & neck 4 clinical	68/52.7	34	2 full	600.8	0/700/75	Moderate
Head & neck 4 complex				1055	1200/2000/100	Off
Head & neck 5 simple				405.2	50/450/100	Very High
Head & neck 5 clinical	68/52.7	34	2 full	562.1	0/700/75	Very Low
Head & neck 5 complex				1217.5	1200/2000/100	Off

* one counter clockwise arc (179°-181°) and one clockwise (181°-179°)

Supplementary Table S4: Treatment plan information for all lung cases. Treatment plans characterized as simple, and complex have been derived from the clinical plan for each case by altering the Monitor Unit (MU) optimization options and the Aperture Shape Controller (ASC) settings.

	Prescribed dose (Gy)	Number of fractions	Arc geometry	Total MU	MU Min/Max/Strength	ASC
Lung 1 simple				350.1	200/350/100	Very High
Lung 1 clinical	68	34	2 halves**	505.1	0/450/50	Moderate
Lung 1 complex				895.3	1000/1500/100	Off
Lung 2 simple				361.9	50/350/100	Very High
Lung 2 clinical	68	34	2 full*	458.4	50/500/75	Very Low
Lung 2 complex				1189.1	900/2000/100	Off
Lung 3 simple				360.8	50/350/100	Very High
Lung 3 clinical	68	34	2 full*	515.7	70/600/75	Moderate
Lung 3 complex				1200.3	1200/2000/100	Off
Lung 4 simple				276.3	50/275/100	Very High
Lung 4 clinical	68	34	2 halves**	325.9	0/400/75	Very High
Lung 4 complex				731.2	800/1200/100	Off
Lung 5 simple	64	32	2 full*	350.7	50/350/100	Very High
Lung 5 clinical				500.1	50/500/75	Very High

Lung 5 complex				1001.3	1000/2000/100	Off

* one counter clockwise arc (179°-181°) and one clockwise (181°-179°)

** one counter clockwise arc (0 -181°) and one clockwise (181°-0)

Supplementary Table S5: Treatment plan information for all gynecological cases. Treatment plans characterized as simple, and complex have been derived from the clinical plan for each case by altering the Monitor Unit (MU) optimization options and the Aperture Shape Controller (ASC) settings.

	Prescribed dose (Gy)	Number of fractions	Arc geometry	Total MU	MU Optimization Min/Max/Strength	ASC
Gynecological 1 simple				452.2	100/450/100	Very High
Gynecological 1 clinical	46.8	26	3 full*	457.9	70/600/75	Very Low
Gynecological 1 complex				1224.6	1200/2000/100	Off
Gynecological 2 simple				475.9	50/450/100	Very High
Gynecological 2 clinical	39	13	2 full**	736.4	50/750/75	Very Low
Gynecological 2 complex				1327.8	1200/2000/100	Off
Gynecological 3 simple				451	100/450/100	Very High
Gynecological 3 clinical	66/51	20	3 full*	600.2	70/600/75	Very Low
Gynecological 3 complex				1252.9	1200/2000/100	Off
Gynecological 4 simple				452.2	50/450/100	Very High
Gynecological 4 clinical	46.8	26	3 full*	592.92	50/560/75	Very Low
Gynecological 4 complex				1144.9	1000/2000/100	Off
Gynecological 5 simple				449.8	50/450/100	Very High
Gynecological 5 clinical	66/51	30	3 full*	551.02	70/600/75	Very Low
Gynecological 5 complex				865	1000/2000/100	Off

* two counter clockwise arc (179°-181°), one clockwise (181°-179°)

** one counter clockwise arc (179°-181°) and one clockwise (181°-179°)
